# Scaling-up an evidence-based intervention for osteoarthritis in real-world settings: a pragmatic evaluation using the RE-AIM framework

**DOI:** 10.1186/s43058-020-00032-6

**Published:** 2020-04-28

**Authors:** Andrew Walker, Annette Boaz, Amber Gibney, Zoe Zambelli, Michael V. Hurley

**Affiliations:** 1 St George’s, University of London and Kingston University, London, UK; 2 Health Innovation Network, London, UK

**Keywords:** Implementation, Scale-up, Sustainability, Complex intervention, Osteoarthritis

## Abstract

**Background:**

Scaling-up and sustaining effective healthcare interventions is essential for improving healthcare; however, relatively little is known about these processes. In addition to quantitative experimental designs, we need approaches that use embedded, observational studies on practice-led, naturally occurring scale-up processes. There are also tensions between having adequately rigorous systems to monitor and evaluate scale-up well that are proportionate and pragmatic in practice. The study investigated the scale-up of an evidence-based complex intervention for knee and hip osteoarthritis (ESCAPE-pain) within ‘real-world’ settings by England’s 15 Academic Health Science Networks (AHSNs).

**Methods:**

A pragmatic evaluation of the scale-up of ESCAPE-pain using the RE-AIM framework to measure Reach, Effectiveness, Adoption, Implementation and Maintenance. The evaluation used routine monitoring data collected from April 2014 to December 2018 as part of a national scale-up programme.

**Results:**

Between 2014 and 2018, ESCAPE-pain was adopted by over 110 clinical and non-clinical sites reaching over 9000 people with osteoarthritis. The programme showed sustained clinical effectiveness (pain, function and quality of life) and high levels of adherence (78.5% completing 75% of the programme) within a range of real-world settings. Seven hundred seventy people (physiotherapists and exercise professionals) have been trained to deliver ESCAPE-pain, and 84.1% of sites have continued to deliver the programme post-implementation.

**Conclusions:**

ESCAPE-pain successfully moved from being an efficacious “research intervention” into an effective intervention within ‘real-world’ clinical and non-clinical community settings. However, scale-up has been a gradual process requiring on-going, dedicated resources over 5 years by a national network of Academic Health Science Networks (AHSNs). Whilst the collection of monitoring and evaluation data is critical in understanding implementation and scale-up, there remain significant challenges in developing systems sufficiently rigorous, proportionate and locally acceptable.

Contributions to the literature

- The study has shown quality and reach can be successfully achieved in a practice-led scale-up process of a complex intervention outside of a controlled study.
- We found RE-AIM a useful framework for investigating scale-up, and we describe how it was operationalised within a pragmatic evaluation.
- The findings provide empirical evidence of the challenges of developing and embedding systems to monitor and evaluate practice-led scale-up in the ‘real world’ that are rigorous *and* pragmatic.

## Background

Scaling-up and sustaining evidence-based health interventions is essential to achieve widespread improvements in the quality of care [1, 2]. However, we have a poor understanding of how effective complex healthcare interventions transition from a trial to being implemented at scale-up and sustained in real-world settings [3–10]. Scale-up needs to be supported by effective monitoring and evaluation systems [4, 11, 12], but there are challenges in balancing adequately rigorous systems to monitor and evaluate scale-up with the need for proportionate and pragmatic approaches [12, 13].

Worldwide, osteoarthritis (OA) is one of the most prevalent causes of pain and disability, and an estimated 6.83 million people consult for knee or hip OA in the UK [14, 15]. Yet current management is largely sub-optimal, and the burden on individuals and society remains high [16–18]. Although rehabilitation professionals (such as physical and occupational therapists) understand the need to implement evidenced-based interventions, their ability to implement new knowledge into clinical practice remains limited [19–21]. ESCAPE-pain is a complex evidence-based intervention (EBI) for people with knee or hip OA that combines structured education and self-management strategies with an individualised exercise regime in line with clinical guidelines [22]. Due to the evidence demonstrating ESCAPE-pain’s clinical and cost effectiveness [23–26], the programme was adopted by England’s 15 Academic Health Science Networks (AHSNs) as a priority for scale-up nationally.

The study aimed to evaluate the scale-up of ESCAPE-pain as a complex EBI by a network of AHSNs in England. Tied to this, we discuss the role of pragmatic data collection and monitoring in efforts to scale EBIs within real-world settings.

## Methods

### Study design

This is a pragmatic evaluation of the scale-up of ESCAPE-pain using the RE-AIM framework [27, 28]. By pragmatic, we refer to an approach that is based in practice (rather than taking a research or theoretical perspective) using routine monitoring data collected as part of an AHSNs’ national programme from April 2014 to December 2018. Table 1 outlines how the RE-AIM framework has been applied within the study to measure Reach, Effectiveness, Adoption, Implementation and Maintenance.

**Table 1:** Mapping the RE-AIM framework to the study

Domain	Description of domain and outcome metric	Outcome measure(s) used
Reach	Individual level measure of participation.	Number of participants and joint affected.
Effectiveness	Participants’ outcome or benefits received.	KOOS/HOOS^ measuring levels of pain, activities of daily living and quality of life.
Adoption	Setting/location programme was adopted.	Type of setting, provider and professional delivering the programme.
Implementation	Factors related to the implementation of the programme.	Number of trained facilitators, facilitator feedback on programme implementation and delivery, self-reported compliance with core components, participant adherence
Maintenance	Whether the programme is maintained (or sustained) post implementation.	Number of sites delivering ESCAPE-pain post-implementation

^Knee/Hip Injury and Osteoarthritis Outcome Score

### ESCAPE-pain programme

ESCAPE-pain is an EBI integrating education and exercise for people with chronic knee and/or hip pain and OA, which promotes self-management to improve quality of life and function [23–26]. People attend in groups of 10–12 people, twice a week, over 6 weeks (12 sessions). Each session is led by a trained facilitator and comprises 20–25 min of structured education about OA and self-management strategies, and 30–45 min of exercise. Details of the programme are available at http://www.escape-pain.org/. ESCAPE-pain is underpinned by a randomised controlled trial and economic evaluation [23–26].

### Scaling-up ESCAPE-pain

NHS England established 15 Academic Health Science Networks (AHSNs) to help accelerate the spread and adoption on innovation in healthcare. In 2014, ESCAPE-pain was selected by the AHSN for south London (Health Innovation Network) as a priority for local scale-up and was resourced by a small team (i.e. 2–3 project managers and administrative support) led by a clinical and programme director. In April 2018, ESCAPE-pain became a national programme for scale-up supported by all 15 AHSNs across England for a 2-year period. Scale-up was coordinated by a national programme manager and dedicated resource (e.g. project manager, clinical champion) within each AHSN to support local scale-up.

### ESCAPE-pain training course

A 1-day training course was developed to support the scale-up of ESCAPE-pain to help ensure fidelity to the core component of the programme and quality. The course is mandatory for anyone delivering ESCAPE-pain. Participants learn about the evidence-base and ethos underpinning ESCAPE-pain, develop a detailed understanding of the programme’s format, and gain skills and knowledge to support the implementation and delivery of the programme.

### Data collection

The AHSNs collect routine data to monitor the scale-up of ESCAPE-pain, which were used to measure outcomes for each domains of the RE-AIM framework. AHSNs receive no participant identifiable data, i.e. providers anonymise all data prior to submitting it.

Reach—The number of participants attending each cohort of ESCAPE-pain and the joint affected (i.e. hip or knee OA). Demographic data are not collected. There are no local prevalence data for hip and knee OA available at the level of individual sites to be able to determine a reliable denominator. Nationally, there are an estimated 4.11 million cases of knee OA and 2.46 million cases of hip OA [29].

Effectiveness—Pre-/post-programme clinical outcomes for participants measured using the Knee/Hip Injury and Osteoarthritis Outcome Score (KOOS and HOOS) sub-scales of pain, activities of daily living (ADL) and quality of life (QoL) [30, 31].

Adoption—The number of sites and the type of setting (e.g. clinical outpatients, non-clinical community), provider organisation (e.g. NHS, local authority/council, charity, leisure/fitness centre) and professional (e.g. physiotherapist, therapy assistant or fitness instructor) delivering ESCAPE-pain.

Implementation—Self-reported compliance with the core components of ESCAPE-pain, namely (i) a 1-h session twice a week for 6 weeks (i.e. 12 sessions), (ii) each session contains exercise and structured education, (iii) the programme follows a cohort structure and (iv) the programme must be delivered by a trained facilitator. Facilitators’ self-reported levels of understand of the programme and ability to implement and deliver the programme via a routine post-training questionnaire. Participant adherence measured by the number of people completing the programme. Completion was defined as participants attending 75% of sessions, to match the level of adherence within the clinical trial [23, 24, 26].

Maintenance—The number of sites continuing or ceasing to deliver ESCAPE-pain at < 1 year, 1–2 years and < 2 years post-implementation. It is not possible to report on maintenance at an individual level because long-term follow-up data for clinical outcomes are not collected.

### Data analysis

Clinical outcome data were available for 3664 people with knee OA from 72 sites and 209 people with hip OA from 33 sites. Only participants with pre- *and* post-outcome data were included in the analysis. Data from all sites were analysed as a single dataset. Paired *t* test was used to determine the mean difference for each subscale, and effect size was calculated using Cohen’s D. Data were analysed using R v3.5.1. Data are presented as mean change in KOOS or HOOS points (confidence intervals, CI), where an increase in scores indicates an improvement. All other data were analysed using descriptive statistics.

## Results

### Reach

**Fig. 1:**
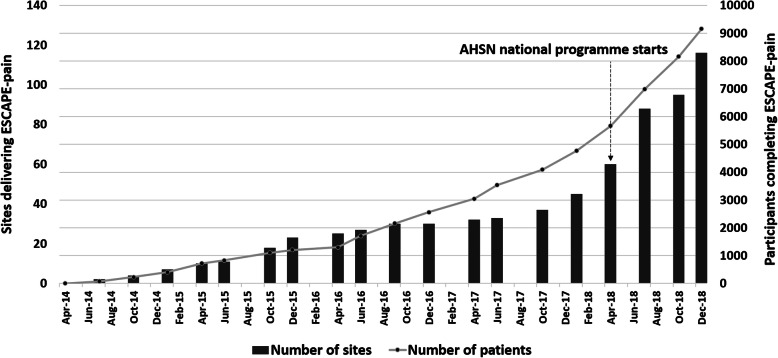
Cumulative number of participants completing and number of sites delivering ESCAPE-pain

Nine thousand one hundred fifty people with hip and knee OA have participated in the ESCAPE-pain programme between April 2014 and December 2018 (Fig. 1).

### Effectiveness

Pre- and post-rehabilitation data were only available from 3614 people with knee and 209 people with hip pain who completed the ESCAPE-pain programme (i.e. defined as attending ≥ 75% of sessions) (Table 2). Participants saw improvements in pain by 7.6 (CI 7.2, 8.1) KOOS points and 5.2 (CI 3.4, 7) HOOS points, function 8.2 (7.7, 8.7) KOOS and 5.5 (3.5, 7.5) HOOS, and quality of life 8.1 (7.5, 8.6) KOOS and 5.6 (3.3, 7.9) HOOS (Table 2). The Western Ontario and McMaster Universities Osteoarthritis Index (WOMAC) function score was used as the primary outcome for the ESCAPE-pain clinical trial [24]. It is possible to calculate the WOMAC function score from the KOOS [31]. WOMAC function score showed an improvement of − 5.49 (95% CI − 7.78, − 3.19, *n* = 278) in the original trial [24], compared to − 5.38 (− 5.69, − 5.06, *n* = 3590) in this study.

**Table 2:** Effectiveness of ESCAPE-pain programme for knee and hip OA

	Sample size^	Pre-mean (SD)	Post-mean (SD)	Mean change (95% CI change)	Effect size (Cohen’s D)
KOOS Domain					
Pain	3614	48.9 (17.3)	56.5 (18.5)	7.6 (7.2, 8.1)**	0.5
Function (ADLs)	3590	53.0 (19.2)	61.1 (20.0)	8.2 (7.7, 8.7)**	0.6
Quality of life	3571	34.0 (18.8)	42.1 (19.8)	8.1 (7.5, 8.6)**	0.5
HOOS Domain					
Pain	209	49.5 (18.4)	54.7(20.5)	5.2 (3.4, 7.0)**	0.4
Function (ADLs)	205	53.7 (20.2)	59.2 (20.8)	5.5 (3.5, 7.5)**	0.4
Quality of life	203	39.7 (21.1)	45.2 (20.5)	5.6 (3.3, 7.9)**	0.3

*KOOS/HOOS* Knee/Hip Injury and Osteoarthritis Outcome Score, *ADLs* activities of daily living, *SD* standard deviation, *CI* confidence interval

^Number of participants with complete datasets (i.e. all sections of the KOOS/HOOS completed before and after completing the programme) out of a total of 9150 people completing the programme

**p < 0.001

### Adoption

Between September 2014 and December 2018, 116 sites were delivering ESCAPE-pain, of which 81 were hospital outpatient departments and 35 in non-clinical community settings (Fig. 1). Following the adoption of ESCAPE-pain as an AHSN national programme in April 2018, there were almost twice the number of sites by December 2018. Compared to the original model of delivery tested within the clinical trial (i.e. physiotherapists within outpatient departments), the programme has been adopted across an increasing range of settings, providers, and profession (Table 3). However, physiotherapists delivering ESCAPE-pain in outpatient departments remain the dominant model (i.e. 70% or 81/116 sites). A comparison of effectiveness by setting type (i.e. clinical versus non-clinical) showed no significant different in outcomes (Table 4), although a smaller dataset was available for non-clinical settings.

**Table 3:** Range of settings, providers and practitioners that have delivered ESCAPE-pain

Setting	Provider	Professional
Physiotherapy dept.	NHS (public health provider)	Physiotherapist
Leisure/fitness centre	NHS (public health provider)	Therapy assistant and/or physiotherapist
Leisure/fitness centre	Leisure/fitness provider	Physiotherapist and/or fitness instructor
Workplace	NHS occupational health	Physiotherapist
Community centre	Third sector	Physiotherapist or fitness instructor
Community centre	Local authority/town council	Physiotherapist and/or fitness instructor

**Table 4:** Effectiveness of ESCAPE-pain programme for knee and hip OA by setting type

	Clinical setting		Non-clinical setting	
	Sample size^	Mean change† (95% CI change)	Sample size^	Mean change† (95% CI change)
KOOS domain				
Pain	3219	7.68 (6.91, 7.93)	395	7.33 (5.86, 8.80)
Function (ADLs)	3196	8.24 (7.35, 8.35)	394	7.74 (6.33, 9.14)
Quality of life	3177	8.11 (7.21,8.39)	394	7.66 (6.02, 9.31)
HOOS domain				
Pain	143	5.40 (3.37, 7.03)	66	4.77 (1.14, 8.40)
Function (ADLs)	140	6.05 (3.53, 7.46)	65	4.30 (0.77, 7.83)
Quality of life	139	6.04 (3.31, 7.87)	64	4.62 (0.35, 8.90)

*KOOS/HOOS* Knee/Hip Injury and Osteoarthritis Outcome Score, *ADLs* activities of daily living, *CI* confidence interval

^Number of participants with complete datasets (i.e. all sections of the KOOS/HOOS completed before and after completing the programme) out of a total of 9150 people completing the programme

†Between-group difference (clinical/non-clinical) for all domains of the HOOS/KOOS was not significant (> 0.05)

### Implementation

Adherence data were available for 6072 participants; 78.5% (*n* = 4767) of participants completed 75% of sessions. Seven hundred seventy people (or facilitators) have been trained to deliver ESCAPE-pain (488 physiotherapists, 282 fitness instructors). Facilitators (*n* = 665) agreed (13%) or strongly agreed (87%) they understood what ESCAPE-pain was and how to implement it, 29% agreed and 71% strongly agreed they felt able to deliver ESCAPE-pain. All sites self-report compliance against the programme’s core 4 components when implementing ESCAPE-pain.

### Maintenance

As of December 2018, 84.1% of sites continue to deliver ESCAPE-pain post-implementation (116 out of a total of 138 sites). Table 5 shows the number of sites delivering/ceasing ESCAPE-pain after the programme was implemented by three time categories (i.e. < 1, 1–2 and > 2 years). Of all the sites that have implemented ESCAPE-pain (*n* = 138), it was stopped after less than 1 year by 11 (8%) sites. Of those sites delivering ESCAPE-pain at December 2018 (*n* = 116), it has been delivered for more than 2 years by 24 (20.7%) sites.

**Table 5:** Number of sites delivering/ceasing ESCAPE-pain post-implementation

Time since implementation (years)*	Number of sites delivering ESCAPE-pain	Number of sites ceasing ESCAPE-pain
< 1	82	11
1–2	10	9
> 2	24	2

*Based on the time the programme began being delivered at a site

## Discussion

The widespread implementation of healthcare innovations usually takes many years; many initiatives fail [3] or are not translated into practice [32]. We used RE-AIM to evaluate the national scale-up of ESCAPE-pain, a complex healthcare intervention for knee and hip OA, by England’s 15 Academic Health Science Networks (AHSNs).

Since 2014, ESCAPE-pain has been adopted in over 110 sites, reaching over 9000 people with knee or hip OA. Whilst this is promising, national prevalence data for knee (4.11 million) and hip (2.46 million) OA in England indicate a need for continuing to expand reach [29]. Although it is delivered predominately by physiotherapists, a growing number of exercise professionals are now delivering the programme. It has been adopted across an expanding range of settings beyond the original model tested in the trial (from NHS outpatient departments to non-clinical community venues), and by a diverse number of providers and funding arrangements. This shows that complex healthcare interventions of this kind can be scaled-up successfully into similar types of settings and professions and ‘spread-out’ into different contexts [3, 5].

Monitoring demonstrates that ESCAPE-pain has been scaled-up; however, it is important to determine what (exactly) has been implemented (i.e. fidelity) and whether it is effective (i.e. delivering intended outcomes) [33, 34]. Measuring intervention fidelity and quality within a national scale-up process has been pragmatic in its approach. A mandatory 1-day training course was developed as a strategy to help safeguard that ESCAPE-pain was implemented with fidelity (i.e. by building knowledge and skills to implement and deliver the programme) [35]. In addition, all sites implementing ESCAPE-pain were required to self-report compliance with the programme’s core components—sites that do not report compliance are not considered to be delivering ESCAPE-pain. Participants’ adherence levels were comparable with those observed within the original trial where 55% of participants completed 10 or more sessions in the ESCAPE-pain group intervention [23, 24].

Individual level long-term follow-up data are not collected as part of the AHSNs’ national programme, which means that it is not possible to determine if the self-management strategies and subsequent benefits delivered by ESCAPE-pain are maintained by participants. At an organisational level, the number of sites continuing to deliver the programme is high, suggesting it is largely sustained in practice settings once implemented. There is debate in the literature about what constitutes sustainability (e.g. continued delivery of intervention components, extent of integration, realisation of outcomes, duration) [8, 34]. In the case of ESCAPE-pain, the majority of sites are < 1-year post-implementation; therefore, the extent of long-term sustainability is to be seen.

As interventions move from highly resourced, controlled research conditions into ‘real-world’ settings, there is a risk effectiveness can be reduced due to the intervention’s essential core components being incorrectly implemented [34, 36]. Therefore, it is important to continue to monitor the effectiveness of interventions as they are implemented in different contexts [12, 13, 34]. Critically for ESCAPE-pain, on-going data collection demonstrated that the programme’s effectiveness has been maintained as it spread from a controlled [24], cloistered trial setting, into very different ‘real-world’ clinical and community settings. However, interpretation of effectiveness needs to recognise the potential impact of missing data as people with poorer outcomes may be underrepresented.

The systematic, on-going monitoring of scale-up demonstrated by AHSNs for ESCAPE-pain is uncommon [12, 13, 34, 37], despite calls to evaluate the widespread implementation of self-management programmes for OA, like ESCAPE-pain [37]. However, data collection has been difficult as staff (both clinical and non-clinical) in sites often lack systems to routinely collect data, have little time and may be unable or reluctant to collect data. Although the AHSNs have created systems to ease the burden of collecting and analysing data, there is no way of enforcing data return. Consequently, data return is sporadic. All sites have returned data, but not all sites return data all the time (i.e. as requested quarterly), and the sites returning data vary overtime. In addition, the completeness of data returned varies. This results in limitations for reporting scale-up.

Other limitations are that implementation outcomes relied on self-reported and indirect measures (e.g. compliance with core components of ESCAPE-pain, numbers trained, facilitators’ ability implement and deliver ESCAPE-pain). However, impartial observation of implementation across a large number of geographically dispersed sites was not feasible. Whilst these measures do not guarantee the programme was implemented and delivered with fidelity or quality, they provided a pragmatic approach to monitoring. A further challenge going forward is that as the number of sites expands it is essential that systems and processes underpinning monitoring (e.g. data collection, quality controls, analysis and reporting) continue to be rigorous and sustainable (i.e. feasibly resourced) [12, 13].

## Conclusions

An evidence-based complex intervention can be implemented at scale successfully: achieving reach *and* maintaining quality. Importantly, ESCAPE-pain’s clinical effectiveness has been sustained as it has transitioned into a diverse range of ‘real-world’ settings, beyond those tested in the original trial. However, scale-up has been a gradual process requiring on-going, dedicated resources over 5 years by a national network of AHSNs.

Whilst the collection of data for monitoring and evaluation is critical in understanding implementation and scale-up, there are significant challenges in developing systems sufficiently rigorous, proportionate and locally acceptable.

## Data Availability

The datasets used and/or analysed during the current study are available from the corresponding author on reasonable request.

## Abbreviations

ADL: Activities of daily living
AHSN: Academic Health Science Network
EBI: Evidence-based intervention
ESCAPE-pain: Enabling Self-management and Coping with Arthritic Pain using Exercise
HOOS: Hip Injury and Osteoarthritis Score
KOOS: Knee Injury and Osteoarthritis Score
OA: Osteoarthritis
QOL: Quality of life
RE-AIM: Reach, Effectiveness, Adoption, Implementation and Maintenance

## References

[1] NHS England. NHS five year forward view. 2014 [cited 2018 Feb 26]. Available from: https://www.england.nhs.uk/wp-content/uploads/2014/10/5yfv-web.pdf.

[2] Horton T, Illingworth J, Warburton W. The spread challenge: how to support the successful uptake of innovations and improvements in health care [Internet]. The Health Foundation; 2018 [cited 2019 Dec 2]. Available from: https://www.health.org.uk/publications/the-spread-challenge.

[3] Ovretveit J (2011). Widespread focused improvement: lessons from international health for spreading specific improvements to health services in high-income countries. Int J Qual Health Care J Int Soc Qual Health Care..

[4] Milat AJ, Bauman A, Redman S (2015). Narrative review of models and success factors for scaling up public health interventions. Implement Sci..

[5] May CR, Johnson M, Finch T (2016). Implementation, context and complexity. Implement Sci..

[6] Barker PM, Reid A, Schall MW (2016). A framework for scaling up health interventions: lessons from large-scale improvement initiatives in Africa. Implement Sci..

[7] Moore JE, Mascarenhas A, Bain J, Straus SE (2017). Developing a comprehensive definition of sustainability. Implement Sci..

[8] Wiltsey Stirman S, Kimberly J, Cook N, Calloway A, Castro F, Charns M (2012). The sustainability of new programs and innovations: a review of the empirical literature and recommendations for future research. Implement Sci..

[9] Greenhalgh T, Robert G, Macfarlane F, Bate P, Kyriakidou O (2004). Diffusion of innovations in service organizations: systematic review and recommendations. Milbank Q..

[10] Lennox L, Maher L, Reed J (2018). Navigating the sustainability landscape: a systematic review of sustainability approaches in healthcare. Implement Sci..

[11] World Health Organisation. Practical guidance for scaling up health service innovations. 2009 [cited 2018 Jan 22]. Available from: http://apps.who.int/iris/bitstream/10665/44180/1/9789241598521_eng.pdf.

[12] Perla RJ, Bradbury E, Gunther-Murphy C (2013). Large-scale improvement initiatives in healthcare: a scan of the literature. J Healthc Qual Off Publ Natl Assoc Healthc Qual..

[13] Davidoff F (2009). Heterogeneity is not always noise: lessons from improvement. JAMA..

[14] Cross M, Smith E, Hoy D, Nolte S, Ackerman I, Fransen M (2014). The global burden of hip and knee osteoarthritis: estimates from the global burden of disease 2010 study. Ann Rheum Dis..

[15] Arthritis Research UK. State of musculoskeletal health 2018: Arthritis and other musculoskeletal conditions in numbers. 2018. Available from: https://www.arthritisresearchuk.org/arthritis-information/data-and-statistics/state-of-musculoskeletal-health.aspx.

[16] Department of Health. CMO annual report: volume one, 2011 ‘On the state of the public’s health’. 2011 [cited 2015 Jan 19]. Available from: http://webarchive.nationalarchives.gov.uk/20130107105354/http://www.dh.gov.uk/health/2012/11/cmo-annual-report/.

[17] Arthritis Research UK. Musculoskeletal health - a public health approach. 2014. Available from: http://www.arthritisresearchuk.org/policy-and-public-affairs/public-health.aspx.

[18] Bishop A, Foster NE, Croft P (2013). SAPC hot topic: is it a dangerous idea to make physiotherapists the gatekeepers of frontline primary care for all patients with musculoskeletal problems?. Prim Health Care Res Dev..

[19] Metcalfe C, Lewin R, Wisher S, Perry S, Bannigan K, Moffett JK (2001). Barriers to implementing the evidence base in four NHS therapies: dietitians, occupational therapists, physiotherapists, speech and language therapists. Physiotherapy..

[20] Harding KE, Porter J, Horne-Thompson A, Donley E, Taylor NF (2014). Not enough time or a low priority? Barriers to evidence-based practice for allied health clinicians. J Contin Educ Health Prof..

[21] Jones CA, Roop SC, Pohar SL, Albrecht L, Scott SD (2015). Translating knowledge in rehabilitation: systematic review. Phys Ther..

[22] NICE. Osteoarthritis: care and management in adults CG 177. NICE; 2014. Available from: http://www.nice.org.uk/guidance/cg177.

[23] Hurley MV, Walsh NE, Mitchell HL, Pimm TJ, Patel A, Williamson E (2007). Clinical effectiveness of a rehabilitation program integrating exercise, self-management, and active coping strategies for chronic knee pain: a cluster randomized trial. Arthritis Rheum..

[24] Hurley MV, Walsh NE, Mitchell H, Nicholas J, Patel A (2012). Long-term outcomes and costs of an integrated rehabilitation program for chronic knee pain: a pragmatic, cluster randomized, controlled trial. Arthritis Care Res..

[25] Jessep SA, Walsh NE, Ratcliffe J, Hurley MV (2009). Long-term clinical benefits and costs of an integrated rehabilitation programme compared with outpatient physiotherapy for chronic knee pain. Physiotherapy..

[26] Hurley MV, Walsh NE, Mitchell HL, Pimm TJ, Williamson E, Jones RH (2007). Economic evaluation of a rehabilitation program integrating exercise, self-management, and active coping strategies for chronic knee pain. Arthritis Rheum..

[27] Glasgow RE, Vogt TM, Boles SM (1999). Evaluating the public health impact of health promotion interventions: the RE-AIM framework. Am J Public Health..

[28] Kessler RS, Purcell EP, Glasgow RE, Klesges LM, Benkeser RM, Peek CJ (2013). What does it mean to “employ” the RE-AIM model?. Eval Health Prof..

[29] Versus Arthritis. Musculoskeletal health 2019: arthritis and other musculoskeletal conditions in numbers. 2019 [cited 2020 Feb 23]. Available from: https://www.versusarthritis.org/media/14594/state-of-musculoskeletal-health-2019.pdf.

[30] Klässbo M, Larsson E, Mannevik E (2003). Hip disability and osteoarthritis outcome score. An extension of the Western Ontario and McMaster Universities Osteoarthritis Index. Scand J Rheumatol..

[31] Roos EM, Lohmander LS (2003). The Knee injury and Osteoarthritis Outcome Score (KOOS): from joint injury to osteoarthritis. Health Qual Life Outcomes..

[32] Grimshaw JM, Eccles MP, Lavis JN, Hill SJ, Squires JE (2012). Knowledge translation of research findings. Implement Sci..

[33] Carroll C, Patterson M, Wood S, Booth A, Rick J, Balain S (2007). A conceptual framework for implementation fidelity. Implement Sci.

[34] Chambers DA, Glasgow RE, Stange KC (2013). The dynamic sustainability framework: addressing the paradox of sustainment amid ongoing change. Implement Sci.

[35] Yin RK (1981). Life histories of innovations: how new practices become routinized. Public Adm Rev..

[36] Dixon-Woods M. Perspectives on context: the problem of context in quality improvement. Health Foundation; 2014 [cited 2016 Feb 20]. Available from: http://www.health.org.uk/publication/perspectives-context.

[37] Allen KD, Choong PF, Davis AM, Dowsey MM, Dziedzic KS, Emery C (2016). Osteoarthritis: models for appropriate care across the disease continuum. Best Pract Res Clin Rheumatol..

